# The next steps

**DOI:** 10.4103/0971-3026.40286

**Published:** 2008-05

**Authors:** Bhavin Jankharia

**Affiliations:** Indian Journal of Radiology and Imaging, Bhaveshwar Vihar, 383 Sardar V P Rd, Prarthana Samaj, Mumbai - 400 004, India. E-mail: editor@ijri.org

The February 2008 issue created history of sorts by being the first issue in many, many years to be published 15 days before its due date, i.e., it was printed and published on 15 January, 2008. This issue too, will be ready on 15 April, 2008 and, hopefully, should be in your hands by the first week of May.

This is just the first step, though. We now need to focus on the quality of the articles and the relevance of the matter that gets published in this journal. As you have seen, we have been publishing small symposia in each issue. This issue is no different; it carries articles on interventional radiology, guest-edited by Dr. Chander Mohan. The August issue will also have a symposium on the same subject, while the November 2008 and January 2009 issues will focus on obstetric ultrasound and the May 2009 and August 2009 issues on breast imaging. Articles on these subjects will be fast-tracked and submissions are welcome.

This issue carries an interesting article on PET/CT in tuberculosis. As you must have noticed, the IJRI is very proactive as far as PET/CT is concerned, and I hope that the IJRI will become the *de facto* voice for all PET/CT-related articles and issues in this country. Contributions related to PET/CT are more than welcome and will be fast-tracked as well.

A new feature in this issue is the series titled ‘Radiology Medicolegal Cases.’ We have taken this report from a journal called ‘*Medical Law Cases - for Doctors*’ and we hope to publish at least one interesting report each quarter. Readers are welcome to send their own contributions as well, related to real cases or their comments and views on these issues.

We are also looking for contributors and those with an editorial flair to help us with ‘Abstracts of Current Literature,’ ‘Excerpts from the Blogosphere,’ ‘Industry News,’ etc. Please e-mail me if you have ideas regarding these or other sections that we can carry in the journal; I will be more than happy to have you on board.

We were hoping that after the last issue, someone somewhere would have been able to locate the very first issue of this journal. Sadly no one has come forth. I refuse to believe that at least one copy of this issue is not lying in one of our senior radiologists' homes or journal collections. I appeal to all radiologists to try and help find the first issue of the IJR (as it was then called). This will be duly acknowledged.

At the risk of sounding repetitive, I appeal to all authors to understand the meaning of plagiarism. As much as 70–80% of all submissions still have significant amounts of copied and pasted material; the commonest excuse the authors give is that they did not know that they are not supposed to copy and paste. Please understand that all articles have to be written in the authors' own words.

Finally, this is the last issue that will carry a radiology quiz. This concept was started sometime in the late 90s and I believe that it has finally run it course. The quizzes that are currently being submitted are typically case reports with a poor quiz quotient; also, the quizzes no longer seem to have any relevance. The August 2008 issue will carry the answer to this issue's quiz and that will be the end of this section.

